# Effect of Oxidation and Silane Modifications Applied to the Bonded Material and Fibers in Carbon-Fiber-Reinforced Composite Adhesive Joints

**DOI:** 10.3390/polym17141893

**Published:** 2025-07-08

**Authors:** Iclal Avinc Akpinar, Ömer Faruk Koçyiğit, Selcuk Atasoy

**Affiliations:** 1 Office of Occupational Health and Safety, Erzurum Technical University, 25050 Erzurum, Turkey; iclal.akpinar@erzurum.edu.tr; 2 Mechanical Engineering, Giresun University, 28200 Giresun, Turkey; faruk.kocyigit@outlook.com.tr

**Keywords:** polymer, oxidation, silane, carbon fiber, adhesive joints

## Abstract

In carbon-fiber-reinforced composites, hydroxyl and carboxyl groups are formed on the carbon fiber surface as a result of the oxidation process applied to the fibers. These groups strengthen the interfacial bond between the fibers and the epoxy resin. In addition, the silanization process chemically bonds amino and glycidyl groups to the fiber surface, further improving adhesion and thus optimizing the performance of the joint. In light of this, the primary objective of the present study is to optimize the performance of adhesive joints by applying oxidation and silane modifications to the fibers added to the adhesive and the bonded metal materials. In this study, carbon fibers underwent oxidation treatment for 5, 10, and 20 min, followed by silanization with 3-aminopropyltriethoxysilane (APTES) and glycidoxypropyltrimethoxysilane (GPTMS) silane agents. Additionally, the surfaces of the bonded aluminum materials were subjected to a 10 min oxidation process, followed by silanization with APTES and GPTMS silane agents. The tensile test performance of single-lap joints, bonded using chemically surface-treated aluminum and composite adhesives containing 2 wt.% chemically treated carbon fibers, was experimentally investigated. According to the contact angle measurement results obtained in this study, aluminum materials subjected to oxidation treatment exhibited superhydrophilic behavior, whereas materials subjected to silanization displayed hydrophilic behavior. A similar trend was observed in the fibers. The performance of adhesive joints increased by approximately 14% when only the aluminum materials underwent oxidation treatment. Moreover, the addition of 2 wt.% carbon fibers to the adhesive enhanced the joint performance by approximately 31%. However, when oxidation treatments of varying durations were applied to both the aluminum materials and the fibers, the joint performance improved by approximately 35% to 40%. When silanization treatments were applied in addition to the oxidation treatments on aluminum and fiber surfaces, the joint performance increased by approximately 68% to 70%. These findings were corroborated through analyses performed using 3D profilometry and Scanning Electron Microscopy (SEM) imaging.

## 1. Introduction

Due to their lightweight nature combined with high strength and elasticity, carbon fibers are widely utilized in resin-based composites within the aerospace, automotive, construction, and aviation industries. The increasing application of these composites in critical fields has highlighted joining them as a significant challenge. Traditional joining methods, such as bolting and riveting, adversely affect the performance of these structural materials due to fiber cutting, matrix damage, and high stress concentrations around drilled holes [1]. However, bonding these fiber-reinforced composite materials with adhesives effectively addresses the issues associated with traditional joining methods [2,3,4].

Two primary methods are commonly employed to enhance the performance of adhesive-bonded joints. The first method involves modifying the overlap length, adhesive thickness, or joint geometry. The second method entails incorporating micro- or nanoscale reinforcement materials into the epoxy-based adhesive. Studies in the literature on the addition of nanostructures to adhesives predominantly focus on incorporating materials such as graphene, carbon, clay, titanium, alumina, and similar nanostructures into epoxy matrices [5,6,7,8,9]. In a study conducted by Hadjez et al. [6], it was observed that incorporating 2 wt.% graphene nanostructures into the adhesive increased the failure load of single-lap joints. This enhancement was found to vary depending on the properties of the adhesive and the structure of the nanomaterial. Furthermore, it was noted that the addition of nanostructures improved the displacement capacity of the adhesive joints. Another advantage of incorporating nanostructures is their ability to enhance the damage-damping capacity, which significantly increased the failure load of the joint. In a study conducted by Akpinar et al. [7], the thermal cycling performance of composite joints was investigated by incorporating 1 wt.% graphene-COOH, carbon nanotube-COOH, and fullerene C60 nanostructures into the adhesive. The findings revealed that when adhesive joints without nanostructures were subjected to thermal cycling, their failure loads decreased significantly. However, for joints bonded with nanostructure-enhanced adhesives, the failure loads increased by approximately 3% to 5% under thermal cycling conditions. While the addition of these micro- or nanoscale reinforcement materials positively influenced the strength of adhesive joints, it was also observed that this effect was not at the desired level, as the added materials could not prevent the propagation of microcracks formed in the adhesive during loading.

In recent years, inspired by the superior performance of composite materials obtained by combining resin with fibers, literature studies have shown the incorporation of various types of fibers into epoxy-based adhesives to enhance the performance of adhesive joints [10,11,12,13]. Although nanoscale carbon fibers [10,11,12,13] have been shown to improve the epoxy adhesive performance, our study focuses on carbon fibers with larger dimensions (7–8 μm thickness and 60 mm length). This distinction is critical because the behavior and interactions of these fibers with the matrix differ significantly from nanoscale fibers. The weak interfacial state caused by the surface inertia of carbon fibers, which possess a high elastic modulus, limits the interfacial bonding with the resin or epoxy matrix. Chemical surface treatments are applied to fibers to enhance their interfacial interaction with epoxy [14,15,16,17]. In a study conducted by Roseno et al. [18], the performance of carbon fiber composites produced with epoxy resin for biomedical applications was investigated. The surfaces of the carbon fibers were oxidized using concentrated nitric acid to improve interfacial bonding, and the fibers were subsequently combined with epoxy resin through a vacuum-assisted resin infusion process. FTIR analysis revealed that the oxidation process generated new chemical functional groups on the carbon fiber surfaces. Composites fabricated with oxidized carbon fibers exhibited higher tensile strength compared to those with non-oxidized fibers, with the tensile strength increasing as the oxidation duration was extended. In a study conducted by Liu et al. [19], an electrochemical oxidation process was applied to improve both the tensile strength of polyacrylonitrile-based carbon fibers and their interfacial bonding strength with the resin matrix. While the chemical elements and functional groups on the surface of the fibers were analyzed by X-ray photoelectron spectroscopy (XPS), the crystal structures of the fibers were analyzed by a Rigaku D/max 2500 X-ray diffractometer (XRD) (Tokyo, Japan) and Renishaw RM2000 Raman spectrometer (Raman) (New Mills, UK). The results indicated that the proposed treatment conditions enhanced the tensile strength of the carbon fibers by 17.1% and improved the interlaminar shear strength by 14.5%. This improvement was attributed to the increased surface roughness and the interaction of oxygen- and nitrogen-containing functional groups on the carbon fiber surfaces.

In addition, the electrochemical oxidation treatment applied to increase the interfacial interaction of fibers with resin or epoxy improves the surface roughness of carbon fibers and leads to improved resin–fiber interfacial bonding by introducing functional groups such as hydroxyl (-OH) and carboxyl (-COOH). Similarly, silanization with APTES or GPTMS agents further improves the interfacial adhesion by introducing amino and glycidyl groups that form chemical bonds with the epoxy matrix [20]. In a study conducted by Wen et al. [21], polyacrylonitrile-based carbon fibers underwent electrochemical oxidation followed by grafting with the KH550 silane coupling agent. The effects of the surface treatment process on the physico-chemical structure of the fiber surface were examined using infrared spectroscopy (IR), X-ray photoelectron spectroscopy (XPS), Raman spectroscopy, and surface tension/dynamic contact angle measurements. The results indicate that the amount of oxygen-containing functional groups on the carbon fiber surface significantly increased after surface treatment. However, the crystalline structure remained largely unchanged following the grafting process with KH550 agents. Furthermore, the combination of electrochemical oxidation and grafting with KH550 silane agents notably enhanced the interlaminar shear strength of the composites compared to untreated fibers.

To enhance the surface area of the bonded metal material, chemical etching with a sodium hydroxide (NaOH) solution and anodizing surface treatments using various solutions are applied. These chemical methods primarily aim to remove oxide layers from the surface of aluminum components and to introduce surface roughness, thereby improving the bonding strength [22,23,24]. In a study conducted by Yiwei et al. [25], the performance of adhesive joints formed using aluminum alloy sheets subjected to phosphoric acid anodization (PAA) under different parameters was investigated. The PAA process resulted in uniform pits and nanoscale pores, which contributed to changes in the surface energy and surface roughness, as observed through the morphology of the interface. The adhesion strength was characterized using lap-shear tensile testing, revealing two distinct epoxy wetting modes. The visible surface energy plays a crucial role in improving adhesive bonding under complete wetting conditions. On the other hand, the roughness value has been shown to significantly affect the adhesion strength in incomplete wetting conditions. As a result, when the visible surface energy is 84.62 mJ/m^2^ and the roughness value is 0.720 μm, the interfacial adhesion strength of the bond is found to be 52.45 MPa.

The findings from the literature review indicate that the application of chemical surface treatments to fibers or electrochemical oxidation processes on the bonded structural materials has a significant impact on the performance of fiber-reinforced composites. This study is one of the first to systematically investigate the combined effects of oxidation duration and silane modifications on both the aluminum adherend surfaces and the carbon fibers incorporated into the adhesive. By optimizing these chemical treatments, this work provides a novel approach to significantly enhancing the mechanical performance of adhesive joints, which has not been comprehensively addressed in prior studies. In the present study, in order to enhance the performance of adhesive joints bonded with epoxy-based adhesives, oxidation treatments were applied to the carbon fibers added to the adhesive for durations of 5, 10, and 20 min, followed by silanization treatments using 3-aminopropyltriethoxysilane (APTES) and glycidoxypropyltrimethoxysilane (GPTMS) silane agents. Additionally, the surfaces of the bonded aluminum materials underwent a 10 min oxidation treatment, followed by silanization with APTES and GPTMS silane agents. The tensile test performance of single-lap joints bonded with chemically surface-treated composite adhesives containing 2% by weight carbon fiber, subjected to various chemical surface treatments, was experimentally investigated. The experimental results obtained were verified through analysis using a 3D profilometer and Scanning Electron Microscopy (SEM) images.

## 2. Material and Methods

### 2.1. Materials

In the presented study, chopped carbon fibers with a thickness of 7–8 µm and a length of 60 mm were used as the fiber added to the adhesive. These fibers were obtained from Composite.net Turkey. The adhesive used was the epoxy-based two-component DP460 structural adhesive, commonly utilized in various structural sectors, including aerospace, automotive, and shipbuilding. This adhesive was purchased from Ege-Bant, the distributor of 3M Kocaeli, Turkey. The bonded material consisted of 6 mm thick AA2024-T3 aluminum alloy plates, which were supplied by Seykoç Aluminum, Ankara, Turkey. Additionally, the chemicals used at different stages of this study (99% purity Acetone (C_3_H_6_O), 95% purity Sulfuric Acid (H_2_SO_4_), 57% purity Nitric Acid (HNO_3_), 99% purity Ethanol (C_2_H_4_O), and Sodium Hydroxide (NaOH)) were supplied by Interlab (İstanbul, Turkey), the Sigma-Aldrich representative in Turkey. 3-Aminopropyltriethoxysilane (APTES) with 97% purity and Glycidoxypropyltrimethoxysilane (GPTMS) with 98% purity were donated by Safic-Alcan, İstanbul, Turkey. Table 1 shows the mechanical properties of the adhesive, adherend, and carbon fiber used in this study. Also, Table 2 shows the chemical composition of the adherend AA2024-T3 aluminum alloy.

### 2.2. Application of Chemical Surface Treatments

**Figure 1:**
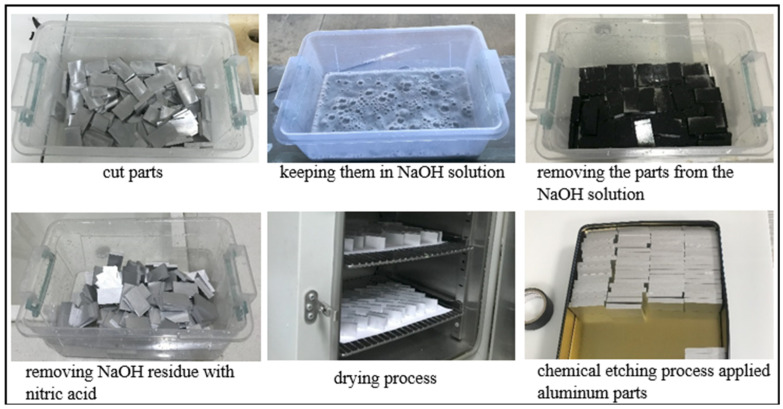
Applying the chemical etching process to aluminum parts.

In the presented study, electrochemical oxidation was applied to the overlap regions of the adherend materials. Prior to this process, a chemical etching procedure was performed to remove contaminants such as dirt, oil, and rust from the surface of aluminum parts with a length of 42.5 mm, a width of 25 mm, and a thickness of 6 mm (Figure 1). For this process, a 15% NaOH solution, made from sodium hydroxide pellets, was used, and the parts were soaked in the NaOH solution for 10 min and then cleaned with distilled water. To remove the NaOH residue from the aluminum parts, the parts were soaked in a 70% nitric acid (HNO_3_) solution for about 2 min, after which the HNO_3_ solution was removed with distilled water [24]. Subsequently, the aluminum parts were dried in an oven at 100 °C for 15 min. In this way, the chemical etching process applied to the aluminum parts was completed, and these parts, ready for the electrochemical oxidation process, were referred to as A_e_ (Figure 1).

**Figure 2:**
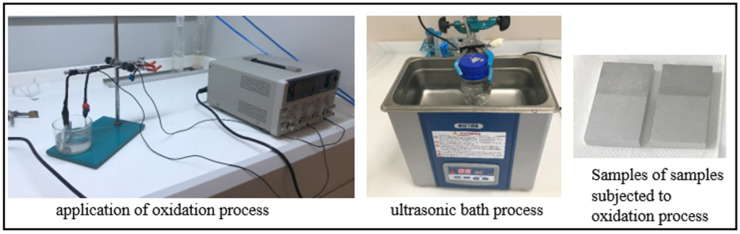
Applying electrochemical oxidation process to aluminum parts.

Electrochemical oxidation was applied to the overlap regions of the aluminum parts that underwent chemical etching. In the literature, the acid solutions commonly used for oxidation are H_2_SO_4_ and HNO_3_, and it is known that these solutions increase the oxygen-containing functional groups on the carbon fiber surface. In the oxidation process, a sulfuric acid (H_2_SO_4_) solution with a concentration of 20% by weight was first prepared as the electrolyte. The aluminum part was placed in the anode region of the device, and a platinum rod was placed in the cathode region. The electrolysis voltage was set to 30 V, the current was approximately 2 A, the duration was 10 min, and the distance between the anode and cathode was set to 40 mm (Figure 2). After the electrochemical oxidation process, the aluminum parts were placed in an empty beaker and cleaned with deionized water. To remove the H_2_SO_4_ solution from the aluminum parts, they were immersed in a 20%-by-weight nitric acid solution in an ultrasonic bath for 1 min (Figure 2). The aluminum parts removed from the nitric acid solution were washed with deionized water and quickly dried with a hairdryer to prevent any stains on the surface. These parts subjected to oxidation were labeled as A_o_.

Before the oxidation process was applied to the carbon fibers, the 60 mm long carbon fibers were mechanically washed in a beaker containing acetone for 20 min. The fibers, removed from the acetone, were dried in an oven at 100 °C for 2 h and then cut to a length of 10 mm. These cleaned fibers were labeled as CF_c_.

**Figure 3:**
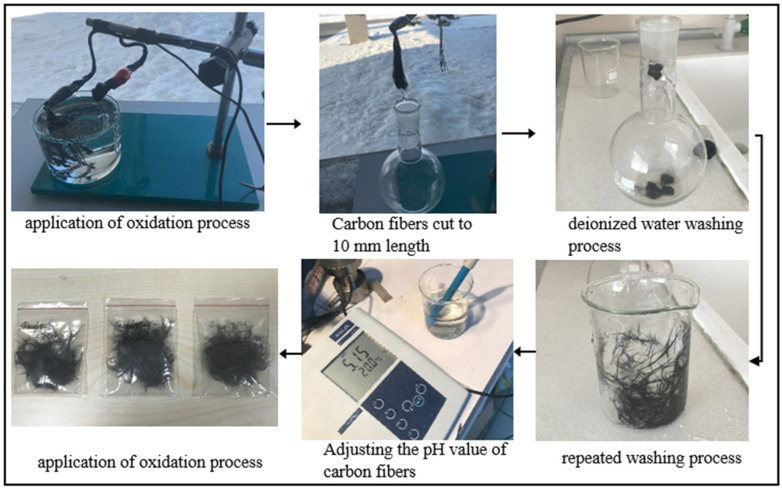
Applying electrochemical oxidation process to carbon fibers.

In addition, the cleaned CF_c_ fibers were subjected to electrochemical oxidation for three different durations (Figure 3). In the oxidation process, sulfuric acid (H_2_SO_4_) with a 50% concentration by weight was used as the electrolyte solution, 60 mm long CF_c_ fibers were used as the anode, and a platinum rod was used as the cathode. The electrolysis voltage was set to 10 V, the current was approximately 1 A, and the distance between the anode and cathode was set to 40 mm. The oxidation process was performed at room temperature for 5, 10, and 20 min. These parameters selected for current and voltage are the values specified as optimal for carbon fiber oxidation. The effect of the oxidation time (5, 10, and 20 min) was determined as a variable to understand how the formation of functional groups on the fiber surface changes over time. After the electrochemical oxidation process, the carbon fibers were cut to a 10 mm length with scissors and a repeated washing process was carried out until the pH value of the oxidized carbon fibers reached approximately 5–6 (Figure 3). The fibers were then dried in an oven at 100 °C for 2 h and the fibers subjected to 5, 10, and 20 min of oxidation were named CF_o/5_, CF_o/10_, and CF_o/20_, respectively. The purpose of the electrochemical oxidation process applied to the carbon fibers for different durations is to improve the interfacial interaction between the epoxy and the fiber by creating functional groups on the surface and to evaluate the relationship between this interaction and the oxidation time.

**Figure 4:**
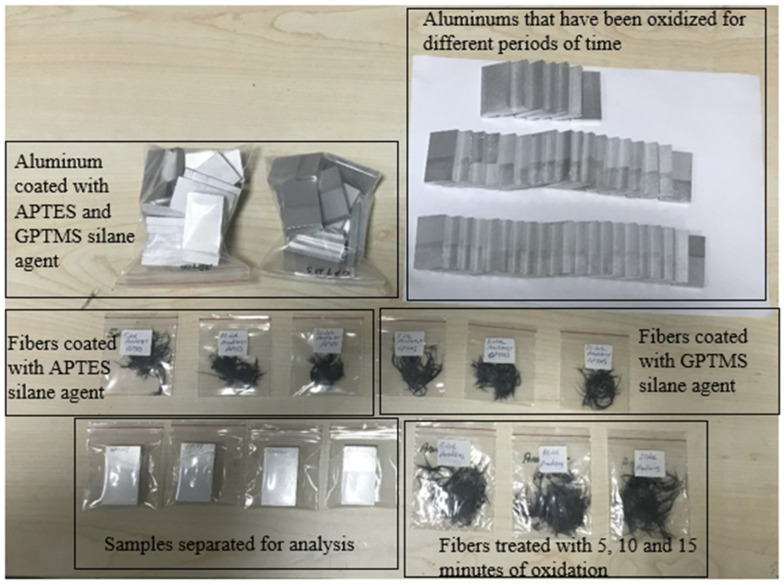
Samples with oxidation and silanization applied to aluminum and carbon fibers before adhesive bonding.

After the electrochemical oxidation process, the surface of the carbon fibers was coated with 3-aminopropyltriethoxysilane (APTES) and glycidoxypropyltrimethoxysilane (GPTMS) silane agents. In order to ensure that the silane coating process was applied homogeneously to the carbon fiber surface, a specific protocol was followed during the process. In this process, 80 g of ethanol (C_2_H_4_O) and 2%-by-weight (1.6 g) APTES were added to an empty beaker to prepare the solution. Then, 0.5 g of GPTMS was added and the solution was mechanically stirred for 10 min using a glass rod (Figure 3). This mixing process was carried out to guarantee a homogeneous distribution of the silane agents in the solution. Then, the carbon fibers were completely immersed in this solution and the solution was allowed to contact the entire fiber surface. After the oxidation process, the carbon fibers were washed several times with deionized water to remove any residual acid until the pH of the washing solution reached a value of approximately 5–6. These fibers, coated with silane agents, were dried in an oven at 100 °C for 2 h. After 5, 10, and 20 min of oxidation, the carbon fibers coated with the APTES silane agent were named CF_o/5A_, CF_o/10A_, and CF_o/20A_, respectively. Additionally, the carbon fibers obtained using GPTMS as the silane agent in the same procedure were named CF_o/5G_, CF_o/10G_, and CF_o/20G_ (Figure 4).

Additionally, the silanization procedure applied to the carbon fibers was also applied to the bonding regions of aluminum materials using the same silane agents. After the electrochemical oxidation process applied to the aluminum, the aluminum pieces coated with the APTES silane agent were named A_o/A_, while the aluminum pieces coated with the GPTMS silane agent were named A_o/G_.

### 2.3. Characterization of Surface-Treated Carbon Fibers and Aluminum Parts

To examine the wettability of the bonded materials and carbon fibers and to calculate the interfacial energy, contact angle tests were conducted. These tests were performed using a Dropmeter A-100p (Ningbo Haishumaishi Testing Co., Ltd., Asia-Pacific, Dongguan City, China) device at room temperature (RT, 24 ± 0.5 °C). In the contact angle test, deionized water (free surface energy $\gamma_{LV}$ = 72.8 mJ/m^2^) was used as the liquid, with a droplet size of 5 μL and a droplet dwell time of 1 s. Measurements were taken from three different points on the samples, and the average value was considered. Based on the contact angles obtained from the test results, the surface contact angle was determined using the Young–Laplace equation. In a study conducted by Baldan [26], it was stated that the contact angle of the liquid on the solid surface is directly related to the surface energies, and this relationship can be associated with the work of adhesion. Therefore, in this study, the work of adhesion is determined using the equation$\;W_{A}=\gamma_{LV}(1+\cos\theta )$, where *θ* is the average contact angle obtained after the test.

Additionally, the surface morphologies of the fibers after electrochemical and silanization treatments applied to the aluminum parts and carbon fibers were observed using a scanning electron microscope (SEM FEI-Quanta 250, Eindhoven, The Netherlands). Furthermore, the fracture morphologies of the bulk samples after testing were also analyzed using SEM. The SEM images taken from the aluminum surface were captured at magnifications ranging from 500× to 8000× for the aluminum parts and from 5000× to 20,000× for the carbon fibers.

To evaluate the surface roughness formed after electrochemical oxidation and silanization coating, surface topographies taken from aluminum samples with dimensions of 1.2 mm in the x-direction, 0.80 mm in the y-direction, and 0.050 mm in the z-direction were analyzed using a 3D optical profilometer device (Bruker Contour GT-I Optical Microscope) (Bruker, Istanbul, Turkey). The surface arithmetic average roughness value (Ra) and the arithmetic average height from the peak to valley (Rz) parameters were obtained through a differential inductance-sensitive roughness detector.

### 2.4. Single-Lap Joint Production

**Figure 5:**
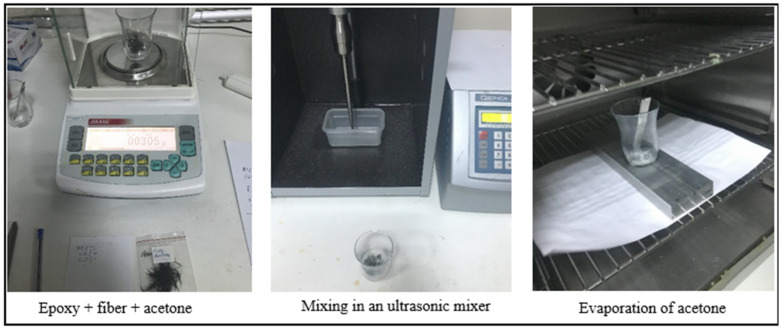
Schematic representation of the method of adding carbon fiber to the adhesive.

In the presented study, the addition of chemically modified and unmodified carbon fibers to the epoxy-based adhesive was determined to be 2% by weight. One of the problems frequently encountered in the literature in the addition of nanostructures and fibers to epoxy adhesives is the homogeneous distribution of nanostructures in the epoxy. The agglomeration of these structures in the epoxy significantly affects the standard deviation in experimental studies. However, in a study conducted by Gültekin et al. [27] in recent years, several different methods were used in the addition of nanostructures to epoxy adhesives, and a new method that reduced the standard deviation to 1% was introduced to the literature. This method was later used by other researchers [28,29,30] and this method was taken into account in the addition of fibers to the adhesive in this study. Considering that 3 adhesive bonding samples were produced for each parameter, the amount of adhesive for 1 parameter was determined to be 3 g. To prepare this, 2 g of epoxy resin was placed in an empty beaker and 0.09 g of carbon fiber (2% by weight) was added. Then, to ensure the homogeneous dispersion and wetting of the fibers within the epoxy, approximately 3 g of acetone were added to the beaker containing the epoxy and fibers, and the mixture was stirred for 10 min using an ultrasonic mixer with a frequency of 30 kHz. After this step, mechanical stirring was performed at 50 °C to evaporate the acetone. Once the complete evaporation of acetone was confirmed using a precise balance, 1 g of hardener was added to the beaker containing the epoxy and fibers, and the mixture was mechanically stirred for 5 min to complete the process (Figure 5).

**Figure 6:**
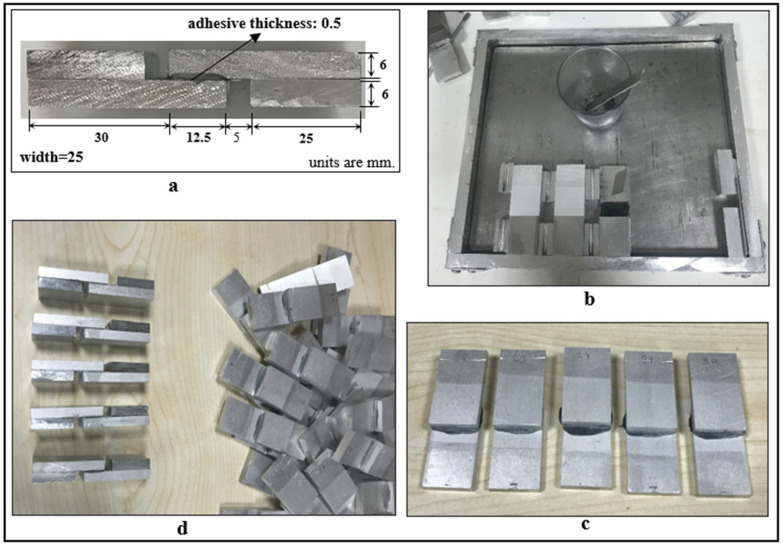
Examples of single-lap joint samples produced: (**a**) geometric dimensions of the single-lap joint, (**b**) single-lap joint production mold, (**c**) single-lap joint samples that have completed curing, and (**d**) single-lap joint samples made suitable for testing.

In the production of single-lap joints bonded with the adhesive, as shown in Figure 6a, the manufacturing mold shown in Figure 6b was used. The adhesive was applied to the overlap regions of the aluminum parts with the help of a spacer. To achieve a 0.5 mm adhesive thickness, metal parts were placed at the free ends of the samples, and aluminum molds of the sample thickness were placed to adjust the overlap length of the samples (Figure 6b). For the curing of the epoxy-based DP460 structural adhesive used in this study, it was kept in an oven at 70 °C for 120 min. After curing, excess adhesive spilling from the joint was removed using cutting tools (Figure 6c). Then, aluminum pieces with dimensions of 25 × 25 mm and the thickness of the bonded material were attached to the free ends of the bonded joints using super glue (Figure 6d). The single-lap joint length used in the present study is shorter than the joint length used in the literature. The reason for choosing a shorter joint length and a thicker bonded material is to minimize the peeling stresses that occur at the ends of the overlap region of the single-lap joints due to eccentric loading.

According to the characteristics of the parameters used in this experimental study, three specimens were produced for each parameter, and each parameter was assigned a code. A total of 72 specimens were produced, with the coding and parameter characteristics shown in Table 3, and these specimens were subjected to tensile tests.

### 2.5. Mechanical Properties of Sing-Lap Joints

**Figure 7:**
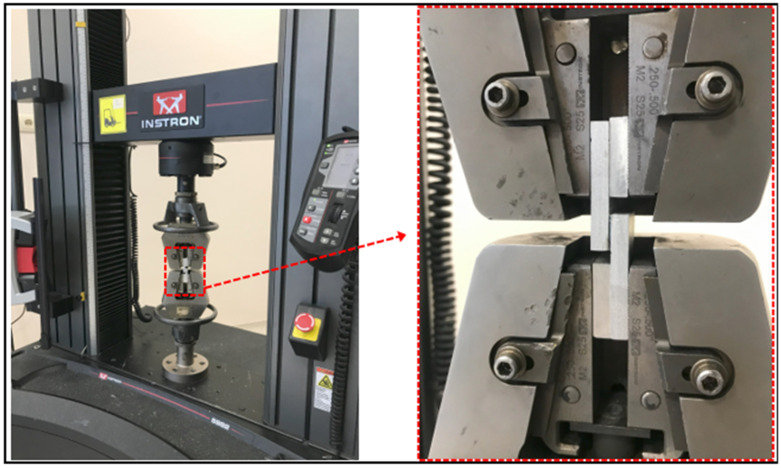
Application of tensile test of single-lap joint.

Tensile tests of single-lap joints bonded with a fiber structure without additives and fiber structure additives with different chemical surface treatments were performed using a computer-controlled Instron-5982 (Norwood, MA, USA) universal tensile testing machine at a tensile test speed of 1 mm/min (Figure 7). Before the test, the overlap length and adhesive thickness of each joint specimen were measured. After the test, the average failure loads and failure surfaces of the joints were analyzed.

## 3. Results and Discussion

### 3.1. Surface Morphologies of Carbon Fibers

**Figure 8:**
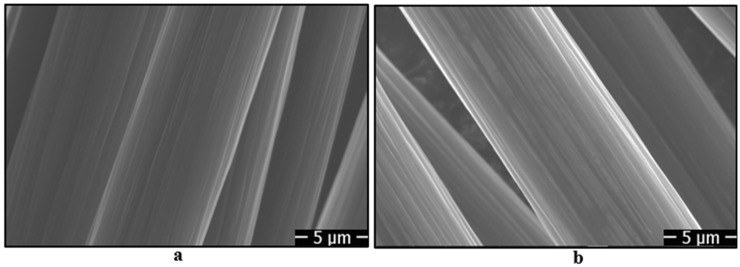

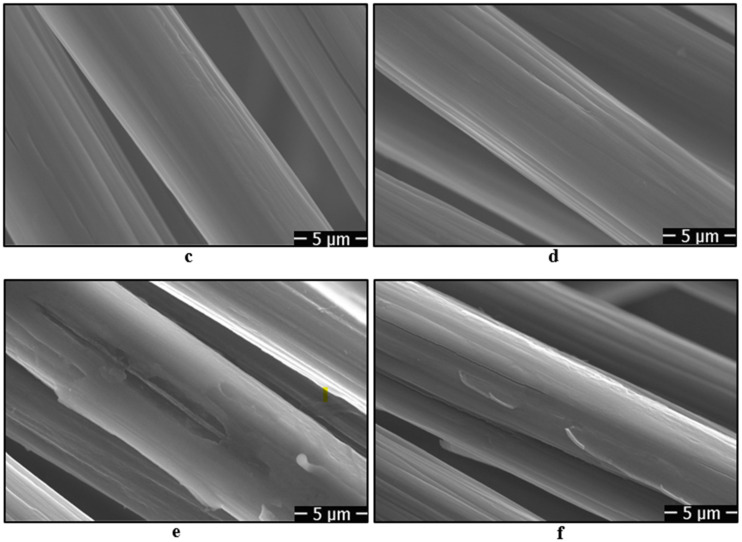
SEM images of carbon fibers: (**a**) cleaned with acetone, (**b**) 5 min oxidation, (**c**) 10 min oxidation, (**d**) 20 min oxidation, (**e**) 10 min oxidation and coating with APTES, and (**f**) 10 min oxidation and coating with GPTMS.

Before adding carbon fibers to the epoxy-based adhesive, oxidation processes were applied to the carbon fibers for different durations, followed by coating with silane agents. When the fibers were cleaned with acetone, SEM images show that there are longitudinal fine grooves on the fiber surface (Figure 8a). However, after the oxidation process, changes in the fine grooves on the fiber surface were observed. Initially, the grooves became more prominent after 5 min of oxidation, likely due to localized etching. With longer oxidation durations (10 and 20 min), the grooves appear to smooth out, indicating the formation of a more uniform surface morphology (Figure 8b–d). This progression confirms the successful implementation of the oxidation process. After the electrochemical oxidation processes applied to the fibers, silanization was carried out using 3-aminopropyltriethoxysilane (APTES) and glycidoxypropyltrimethoxysilane (GPTMS) agents. Upon examining the SEM images given in Figure 8e,f, it is observed that the fiber surfaces are coated with a thin film layer of silane agents, thereby altering the fiber’s topography.

### 3.2. Surface Roughness

**Figure 9:**
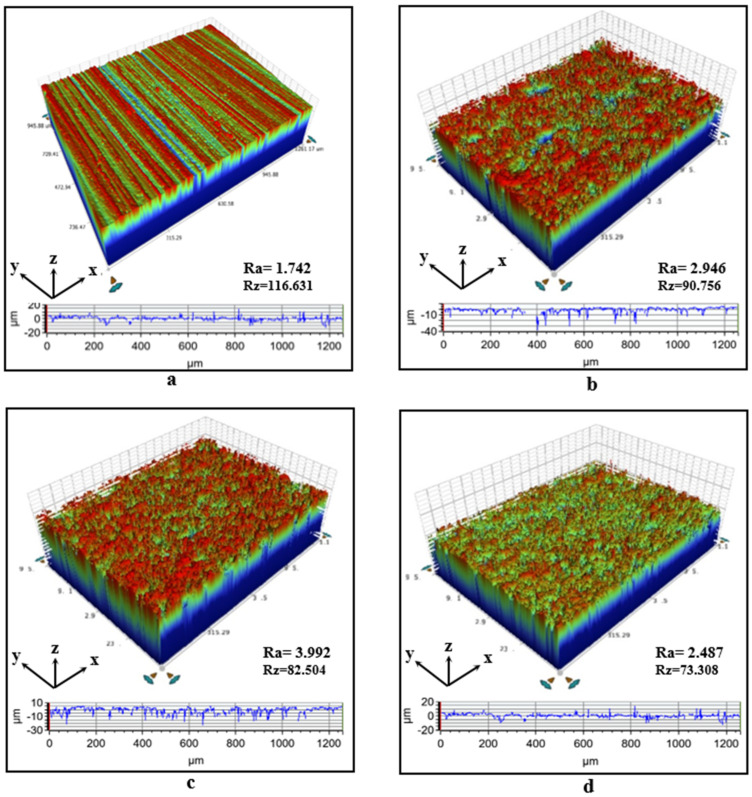
Three-dimensional topography images of aluminum adherends with different treatments: (**a**) the surface was cleaned with sandpaper, (**b**) chemical etching with NaOH, (**c**) oxidation, and (**d**) oxidation and APTES.

In the presented study, the bonded aluminum materials were first cleaned of contaminants on the aluminum surface with 240 SiC sandpaper, and the roughness degree on the surfaces of the aluminum parts after the process is given in Figure 9a. Then, a chemical etching process was applied with NaOH solution, and the micro-hole depth distribution and roughness degree on the surfaces of the aluminum parts after the chemical etching process are given in Figure 9b. Upon examining this surface profile, the arithmetic average surface roughness (Ra) was found to be 2.946 µm and the average peak-to-valley height (Rz) was 90.756 µm. However, after applying anodizing surface treatment for 10 min, the Ra value of the aluminum parts increased by approximately 35%, reaching 3.992 µm, as shown in Figure 9b. After the anodizing surface treatment was applied to the aluminum parts, when the surface was coated with APTES agent, the arithmetic average surface roughness (Ra) decreased to 2.487 µm. This indicates that the surface has been effectively coated with the silane agent. Furthermore, the decrease in the arithmetic mean (Rz) value from peak to valley after electrochemical treatment can be explained by the filling of some of the micro-holes or valleys on the aluminum surface by oxide layers formed during the anodizing process.

An important point to note is that the roughness on the surface of the adherend material will facilitate mechanical interlocking between the adhesive and the adherend surfaces, significantly enhancing the bond strength. However, the anodizing treatment applied to the aluminum surface plays a crucial role in homogenizing the surface roughness and forming concentrated peaks and valleys, which are important for the bond strength. On the other hand, excessively high roughness values could negatively impact the bond strength by reducing the adhesive’s ability to wet the surface.

### 3.3. Contact Angle Measurements

The contact angle varies depending on the properties of the solid and liquid, influencing the surface wettability and adhesion performance. In this study, the contact angle values were used to evaluate the effect of surface treatments on wettability and adhesion.

**Figure 10:**
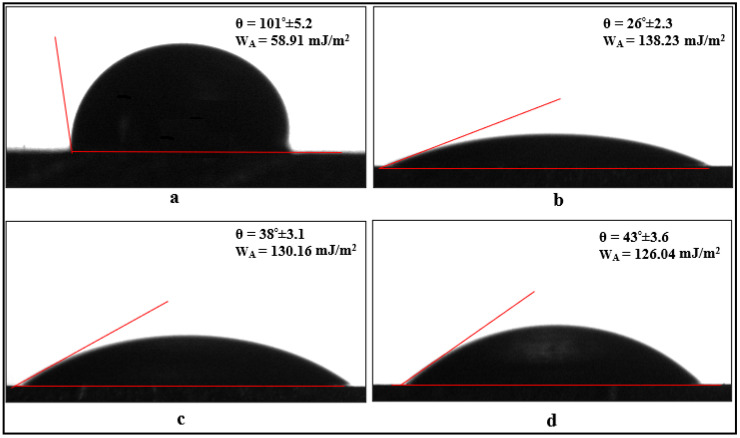
Contact angles corresponding to water drops applied to aluminum parts: (**a**) cleaned with chemical etching, (**b**) oxidized for 10 min, (**c**) oxidized and APTES applied, and (**d**) oxidized and GPTMS.

Additionally, the contact angle determined by the contact angle method was used in the adhesion work equation to calculate the adhesion work values. The surface of the aluminum parts cleaned by chemical etching was hydrophobic, with a contact angle of approximately 101 degrees (Figure 10a). However, when a 10 min oxidation surface treatment was applied to these aluminum parts, the surface transitioned to a hydrophilic state, and the contact angle was approximately 26 degrees, as seen in Figure 10b. This indicates that the oxidation treatment applied to the aluminum parts significantly improved their wettability. When the oxidized surfaces of these parts were coated with APTES and GPTMS silane agents, the contact angle values increased slightly, reaching approximately 38° and 43° degrees, respectively (Figure 9c,d). The increase in contact angles serves as evidence that the aluminum surfaces have been coated with silane agents. Additionally, considering that the contact angle is related to the adhesion work, it can be concluded that, compared to the parts cleaned by chemical etching, the adhesion work of aluminum surfaces that were roughened by oxidation surface treatment increased by approximately 135% (about 2.3 times). The adhesion work between a liquid and a solid surface is influenced by the solid surface energy, which enhances the attraction between the liquid molecules and the solid surface. As a result, this improves the durability of adhesive joints.

**Figure 11:**
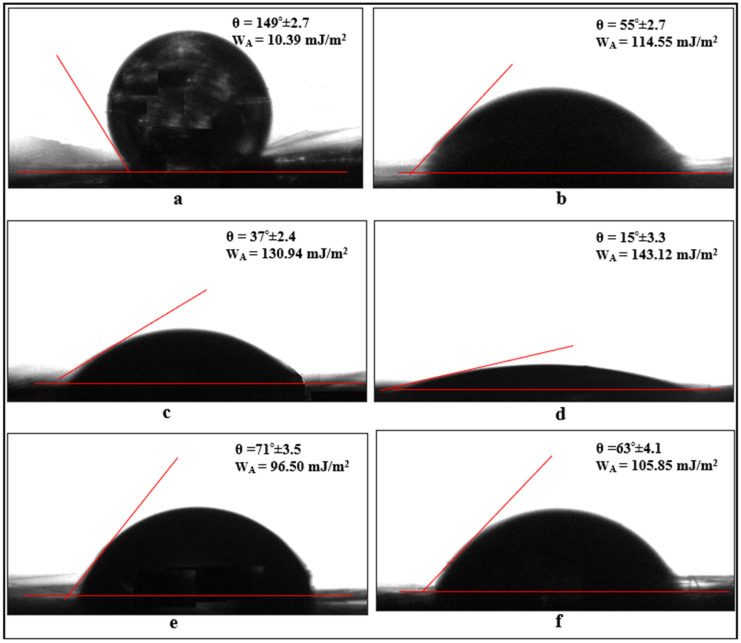
Contact angles corresponding to water drops applied to carbon fibers: (**a**) cleaned with acetone, (**b**) oxidized for 5 min, (**c**) oxidized for 10 min, (**d**) oxidized for 20 min, (**e**) 10 min oxidation and APTES applied, and (**f**) 10 min oxidation and GPTMS applied.

When examining the contact angle values for carbon fibers shown in Figure 11, it is observed that the surface of the fibers cleaned with acetone has a contact angle of 149°, classifying the surface as superhydrophobic (Figure 11a). Upon applying electrochemical oxidation treatment to the carbon fibers for durations of 5, 10, and 20 min, the hydroxyl and carboxyl (-OH and -COOH) groups formed on the fiber surface reduce the contact angle values to 55°, 37°, and 15°, respectively (Figure 11b–d). This indicates that the oxidation treatment applied to the fibers enhances the wettability (hydrophilicity) of the surface, with the wettability changing in relation to the oxidation time.

However, after applying a 10 min electrochemical oxidation treatment to the carbon fibers, the contact angle value is 37°. When the fiber surfaces are coated with APTES and GPTMS agents, the contact angle values increase to an average of 71° and 63°, respectively. An important point here is that the wettability of the fibers, whether very low (superhydrophobic) or very high (superhydrophilic), can negatively affect the interfacial interaction between the epoxy and the fiber. Therefore, the silane agents applied to the fibers help balance the surface wettability. Additionally, considering that contact angles are related to adhesion work, it is observed that applying oxidation treatments to the fibers increases the adhesion work values by approximately 3 to 10 times. When the fibers are coated with silane agents, the adhesion work value increases by approximately two times (Figure 11).

### 3.4. Mechanical Strengths of Single-Lap Joints

**Figure 12:**
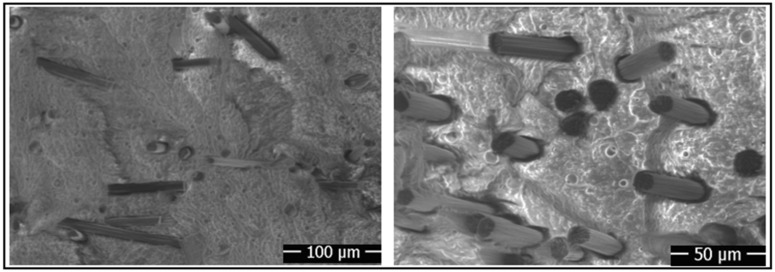
SEM image taken from the surface of carbon-fiber-reinforced composite adhesive.

The average tensile test results of single-lap bonded joints, created by adding 2%-by-weight carbon fibers to the adhesive after chemical surface treatments, are presented in Table 4. The values reported in Table 4 also include the standard deviation (±) calculated from three samples, ensuring statistical reliability. The occurrence of low experimental errors according to the standard deviations is attributed to the meticulous sample preparation protocols used, including the use of acetone and ultrasonic mixing methods to ensure the homogeneous distribution of fibers in the epoxy matrix in the production of composite adhesives (Figure 12), the precise control of the epoxy-hardener ratio in the production of joints, the equalization of the adhesive thickness in all samples, and the preparation of aluminum surfaces. Additionally, the environmental conditions during testing were strictly controlled, minimizing external variations. The average failure strengths given in Table 4 were obtained by dividing the average failure loads obtained from the experiments by the bonding area (12.5 mm × 25 mm).Upon reviewing these test results, it can be observed that the failure strength of the bonded joint with the oxidation-treated aluminum parts (A_o_) increased by approximately 14.4% compared to the carbon-fiber-free connection (A_e_). The oxidation process applied to the aluminum parts increases the surface homogeneity and roughness, which enhances the adhesion work at the solid–liquid interface (improving wettability) and ensures mechanical interlocking. This leads to an increase in the strength of the joint. Furthermore, adding 2%-by-weight carbon fibers to the adhesive along with oxidation-treated aluminum parts (A_o_CF) increases the failure strength of the joint by approximately 30.7%. The reason for the 16.3% increase in failure strength caused solely by the addition of fibers is that the capillary cracks that form in the adhesive during loading and lead to damage are blocked by the fibers.

The application of oxidation processes (A_o_CF_o/5_, A_o_CF_o/10_, and A_o_CF_o/20_) for 5, 10, and 20 min to the carbon fibers added to the adhesive increases the failure strengths of the joint by approximately 34.7%, 38.3%, and 40.2%, respectively. A noteworthy point is that the effect of the oxidation treatment on the failure strength increase ranges from 4% to 10%. Another point to consider is that this increase varies depending on the oxidation time applied to the fibers and the change is not proportional. The increase in failure strength can primarily be attributed to the COOH groups formed on the fiber surfaces and the nanoscale roughness resulting from the electrochemical oxidation of the carbon fibers. Moreover, in addition to the oxidation treatment applied to the fibers, coating the surfaces of the bonded aluminum parts with APTES (A_o/A_CF_o/5_, A_o/A_CF_o/10_, and A_o/A_CF_o/20_) increases the failure strengthby approximately 9%, and coating with GPTMS (A_o/G_CF_o/5_, A_o/G_CF_o/10_, and A_o/G_CF_o/20_) further increases it by 5%. This increase is due to the amino (-NH2) groups in the APTES silane agent chemically reacting with the epoxide rings in the epoxy matrix and forming crosslinks. These crosslinks significantly increase the chemical bonding between the fiber and epoxy. In addition, the glycidyl groups in the GPTMS silane agent react with the epoxy resin and form covalent bonds at the interface. This provides a higher interaction between the epoxy and the fiber interface. However, when the silane coating is applied only to the carbon fibers that have undergone oxidation treatments for different durations (A_o_CF_o/5A_, A_o_CF_o/10A_, and A_o_CF_o/20A_), this increase in the failure strength, which ranges from 34.7% to 40.2%, is further raised to approximately 56.2% to 59.3%. The increase of 20% and 16% in the bond strength due to the application of APTES and GPTMS silane agents to the fibers can be explained by the presence of amino groups formed on the surface by APTES and the glycidyl groups formed by GPTMS, which enhance the interfacial adhesion between the epoxy and the fiber, thus preventing the detachment of fibers from the epoxy.

Additionally, the effects of applying oxidation and silane agents to both the bonded aluminum parts and the fibers added to the adhesive, as well as the application of oxidation and silane agents to the fibers for different durations, on the bond strength have been investigated (Table 4). When this investigation is conducted based on the failure strength of the baseline bond type (A_e_), it is found that the application of oxidation treatments in addition to the APTES silane agent to the surfaces of the bonded aluminum and carbon fibers (A_o/A_CF_o/5A_, A_o/A_CF_o/10A_, and A_o/A_CF_o/20A_) increases the failure strength of the bond by approximately 66.8% to 74.2% (Table 4). When GPTMS is used as a silane agent (A_o/G_CF_o/5G_, A_o/G_CF_o/10G_, and A_o/G_CF_o/20G_), this increase in the failure strength ranges from approximately 61.2% to 66.1% (Table 4). The oxidation treatment applied to the bonded aluminum parts and fiber structures increases the surface energy, facilitating the better spreading and adhesion of liquids to the surface. The application of APTES or GPTMS silane agents to the oxidized aluminum parts and fibers leads to the formation of chemical bonds by the interaction of amino or glycidyl groups with epoxy. As a result, the adhesion of epoxy to both the aluminum surface and the fiber surface is enhanced, significantly increasing the strength of the adhesive bond.

**Figure 13:**
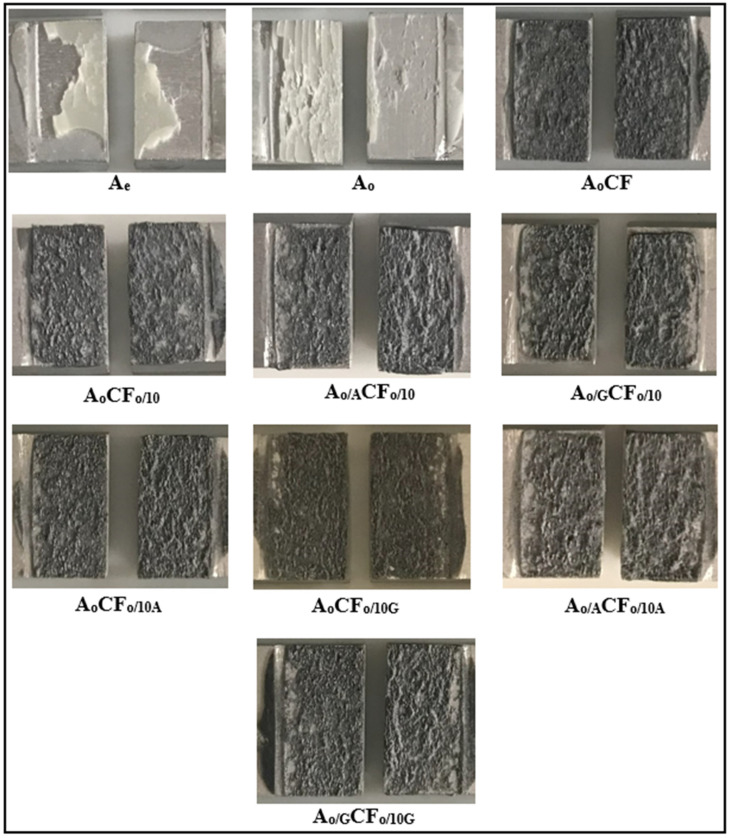
Post-damage surfaces of adhesive joints with pure and different chemical surface treatments of carbon fiber.

In order to better analyze the increase in the failure strength of the adhesive-bonded single-lap joints, the post-damage surface morphologies need to be examined. Therefore, the surface morphologies of the joints after damage, as shown in Figure 13, were evaluated according to the damage modes specified in ISO 10365 [31]. Upon examining these surface morphologies, it was observed that when the aluminum parts were cleaned only by chemical cleaning, adhesive damage (damage at the interface between the adhesive and the bonded material) occurred in the joint, whereas when an oxidation process was applied to the aluminum surface, cohesive damage (damage where the adhesive detaches from the interface) was observed in the joints.

These damage modes demonstrate that the application of oxidation treatment changes the surface energy of aluminum, enhancing the adhesion of epoxy to the surface and increasing mechanical interlocking. Additionally, the incorporation of fibers treated with oxidation or silan processes into the adhesive leads to cohesive damage, transforming the surface morphology into a rough one (Figure 13). This indicates that chemically treated fibers adhere well to epoxy and cannot be peeled off. The damage modes obtained after failure are found to be in strong agreement with the failure strength observed in the joints.

## 4. Conclusions

In the present study, oxidation and silane modifications were applied to carbon fibers (2 wt%) added to the adhesive and the bonded aluminum parts to optimize the performance of the adhesive joints. The results obtained from this study are as follows:

- When the carbon fibers were cleaned with acetone, longitudinal linear micro-grooves were observed on the surface of the fibers. However, when oxidation treatments were applied for different durations, the micro-grooves on the fiber surface were reduced, as observed from SEM images. Additionally, when silanization treatments were applied to the fiber surfaces, a thin film layer formed by the silane agents was observed on the surface.
- The application of oxidation and silanization treatments to the aluminum surfaces increased the surface energy of the aluminum, as revealed by contact angle tests. The adhesion work obtained using contact angle tests showed that the adhesion work of surfaces subjected to oxidation treatment increased by approximately 135%.
- The contact angle on the surface of carbon fibers without chemical surface treatment is 149°. However, when electrochemical oxidation is applied to the fibers for 5, 10, and 20 min, the hydroxyl and carboxyl (-OH and -COOH) groups formed on the fiber surface reduce the contact angle values to 55°, 37°, and 15°, respectively. This indicates that oxidation treatment increases the wettability (hydrophilicity) of the fiber surface, and the wettability changes depending on the oxidation duration. However, when the fiber surface is coated with APTES and GPTMS agents, the contact angle values increase to an average of 71° and 63°, respectively, balancing the surface wettability form with the silane agents.
- The effect of applying oxidation treatments for 5, 10, and 20 min to carbon fibers added to the adhesive results in an increase in the failure strength of the connection by 4% to 10%. However, in addition to the oxidation treatments, coating the fibers with APTES increases this failure strength by approximately 20%, while GPTMS coating increases it by 16%.
- Compared to the failure strength obtained from the basic adhesive connection type without fibers and without chemical surface treatments, the application of oxidation treatments to the aluminum and carbon fiber surfaces, in addition to coating with APTES silane agent, increases the failure strength of the connection by approximately 66.8% to 74.2%. When GPTMS is used as the silane agent; this increase ranges from 61.2% to 66.1%.
- When the aluminum parts’ surface is cleaned only with a chemical degreasing process, adhesive failure occurs at the joint, while the oxidation treatment of the aluminum parts’ surface results in cohesive failure in all joints. These damage modes demonstrate that the oxidation treatment alters the surface energy of the aluminum, improving epoxy adhesion and increasing mechanical interlocking. Additionally, the surface damage modes observed after the test are in strong agreement with the failure strengths obtained from the tensile tests of the joints.

## Figures and Tables

**Table 1:** Mechanical properties of the adherend and adhesive used in this study.

	Carbon Fiber	AA2024-T3	DP 460
σ_t_ (MPa)	3760^±380^	458^±13^	37.2^±0.9^
ε_t_ (%)	1.85	16.2	4.5
ν	-	0.32	0.38
E (MPa)	226,000^±2700^	71,050^±535^	1963^±57^

E: Young’s modulus; ν: Poisson’s ratio; σ_t_: Ultimate tensile strength; ε_t_: Ultimate tensile strain.

**Table 2:** Chemical composition of AA2024-T3 aluminum alloy used in this study.

Element	Cu	Mg	Mn	Fe	Zn	Si	Ti	Others	Al
% by weight	4.48	1.57	0.58	0.17	0.16	0.06	0.03	≤0.04	Remainder

**Table 3:** Parameters used in this experimental study.

Sample Code	Aluminum Surface Treatment	Fiber Surface Treatment	Fiber Ratio (%)
A_e_	chemical etching	-	-
A_o_	oxidation	-	-
A_o_CF	oxidation	cleaned with acetone	2
A_o_CF_o/5_	oxidation	5 min oxidation	2
A_o_CF_o/10_	oxidation	10 min oxidation	2
A_o_CF_o/20_	oxidation	20 min oxidation	2
A_o/A_CF_o/5_	oxidation and APTES	5 min oxidation	2
A_o/A_CF_o/10_	oxidation and APTES	10 min oxidation	2
A_o/A_CF_o/20_	oxidation and APTES	20 min oxidation	2
A_o/G_CF_o/5_	oxidation and GPTMS	5 min oxidation	2
A_o/G_CF_o/10_	oxidation and GPTMS	10 min oxidation	2
A_o/G_CF_o/20_	oxidation and GPTMS	20 min oxidation	2
A_o_CF_o/5A_	oxidation	5 min oxidation and APTES	2
A_o_CF_o/10A_	oxidation	10 min oxidation and APTES	2
A_o_CF_o/20A_	oxidation	20 min oxidation and APTES	2
A_o_CF_o/5G_	oxidation	5 min oxidation and GPTMS	2
A_o_CF_o/10G_	oxidation	10 min oxidation and GPTMS	2
A_o_CF_o/20G_	oxidation	20 min oxidation and GPTMS	2
A_o/A_CF_o/5A_	oxidation and APTES	5 min oxidation and APTES	2
A_o/A_CF_o/10A_	oxidation and APTES	10 min oxidation and APTES	2
A_o/A_CF_o/20A_	oxidation and APTES	20 min oxidation and APTES	2
A_o/G_CF_o/5G_	oxidation and GPTMS	5 min oxidation and GPTMS	2
A_o/G_CF_o/10G_	oxidation and GPTMS	10 min oxidation and GPTMS	2
A_o/G_CF_o/20G_	oxidation and GPTMS	20 min oxidation and GPTMS	2

**Table 4:** Tensile test results of joints (values represent mean failure loads ± standard deviation, based on three specimens per parameter (n = 3)) with different chemical surface modifications.

Sample Code	Average Failure Strenght (MPa)	% Increase Rate
A_e_	21.07 ± 0.43	
A_o_	24.11 ± 0.59	14.4
A_o_CF	27.54 ± 0.87	30.7
A_o_CF_o/5_	28.38 ± 0.60	34.7
A_o_CF_o/10_	29.35 ± 0.46	38.3
A_o_CF_o/20_	29.54 ± 0.66	40.2
A_o/A_CF_o/5_	30.17 ± 0.67	43.2
A_o/A_CF_o/10_	30.99 ± 0.61	47.1
A_o/A_CF_o/20_	31.50 ± 0.56	49.5
A_o/G_CF_o/5_	29.77 ± 0.33	41.3
A_o/G_CF_o/10_	30.45 ± 0.51	44.6
A_o/G_CF_o/20_	30.70 ± 0.71	45.7
A_o_CF_o/5A_	32.91 ± 0.65	56.2
A_o_CF_o/10A_	33.40 ± 0.63	58.5
A_o_CF_o/20A_	33.56 ± 0.61	59.3
A_o_CF_o/5G_	31.98 ± 0.53	51.8
A_o_CF_o/10G_	32.68 ± 0.50	55.1
A_o_CF_o/20G_	33.06 ± 0.67	56.9
A_o/A_CF_o/5A_	35.15 ± 0.74	66.8
A_o/A_CF_o/10A_	36.71 ± 0.66	74.2
A_o/A_CF_o/20A_	35.92 ± 0.59	70.5
A_o/G_CF_o/5G_	33.97 ± 0.55	61.2
A_o/G_CF_o/10G_	34.42 ± 0.69	63.4
A_o/G_CF_o/20G_	34.99 ± 0.61	66.1

## Data Availability

The data presented in this study are available on request from the corresponding author.
